# A Novel Germline Compound Heterozygous Mutation of *BRCA2* Gene Associated With Familial Peripheral Neuroblastic Tumors in Two Siblings

**DOI:** 10.3389/fgene.2021.652718

**Published:** 2021-07-23

**Authors:** Yeran Yang, Jiwei Chen, Hong Qin, Yaqiong Jin, Li Zhang, Shen Yang, Huanmin Wang, Libing Fu, Enyu Hong, Yongbo Yu, Jie Lu, Yan Chang, Xin Ni, Min Xu, Tieliu Shi, Yongli Guo

**Affiliations:** ^1^Beijing Key Laboratory for Pediatric Diseases of Otolaryngology, Head and Neck Surgery, MOE Key Laboratory of Major Diseases in Children, National Center for Children’s Health, Beijing Children’s Hospital, Beijing Pediatric Research Institute, Capital Medical University, Beijing, China; ^2^Biobank for Clinical Data and Samples in Pediatric, National Center for Children’s Health, Beijing Children’s Hospital, Beijing Pediatric Research Institute, Capital Medical University, Beijing, China; ^3^Beijing Advanced Innovation Center for Big Data-Based Precision Medicine, Beihang University, Capital Medical University, Beijing, China; ^4^Center for Bioinformatics and Computational Biology, School of Life Sciences, Institute of Biomedical Sciences, East China Normal University, Shanghai, China; ^5^Department of Surgical Oncology, National Center for Children’s Health, Beijing Children’s Hospital, Capital Medical University, Beijing, China; ^6^Department of Pathology, National Center for Children’s Health, Beijing Children’s Hospital, Capital Medical University, Beijing, China; ^7^Department of Surgery, Shanghai Children’s Medical Center, School of Medicine, Shanghai Jiao Tong University, Shanghai, China

**Keywords:** peripheral neuroblastic tumors, neuroblastoma, ganglioneuroma, whole-genome sequencing, RNA-Seq, *BRCA2*, cancer predisposition gene

## Abstract

**Objectives:**

To investigate the genetic variants that are responsible for peripheral neuroblastic tumors (PNTs) oncogenesis in one family case.

**Materials and Methods:**

One family was recruited, including the healthy parents, sister affected by neuroblastoma (NB), and brother who suffered from ganglioneuroma (GN). Whole-genome sequencing (WGS) of germline DNA from all the family members and RNA-seq of tumor RNA from the siblings were performed. Mutants were validated by Sanger sequencing and co-IP was performed to assess the impact of the mutant on chemosensitivity in the SH-SY5Y cell line.

**Results:**

A novel compound heterozygous mutation of *BRCA2* was locked as the cause of carcinogenesis. One allele was BRCA2-S871X (stop-gain) from the siblings’ mother, the other was BRCA2-N372H (missense) from their father. This novel compound heterozygous mutations of the *BRCA2* gene associated with PNTs by disordering DNA damage and response (DDR) signal pathway. Moreover, chemosensitivity was reduced in the NB cell line due to the BRCA2-N372H mutant.

**Conclusion:**

In summary, these results revealed a novel germline compound heterozygous mutation of the *BRCA2* gene associated with familial PNTs.

## Introduction

Peripheral neuroblastic tumors (PNTs), which account for 7–10% of all tumors in children, arise from primitive sympathogonia (Luksch et al., 2016). The PNTs encompass the histologic variants neuroblastoma (NB), ganglioneuroblastoma (GNB), and ganglioneuroma (GN). As tumors of the sympathetic nervous system, they arise wherever sympathetic tissue exists with a predilection for the adrenal gland and retro peritoneum. The biggest difference among these three tumors is the differentiation of tumor cells and prognosis. NB is the most undifferentiated and malignant tumor of the three. As a rare pediatric cancer, NB affects 10.2 per million children under 15 years of age and accounts for 15% of cancer deaths in children (Cheung and Dyer, 2013). The most benign tumor is GN, which is composed of well-differentiated gangliocytes and mature stroma (Lonergan et al., 2002). A familial history of NB is very rare, which is reported in about 1% of patients (Janoueix-Lerosey et al., 2010). Missense and frameshift mutations of *PHOX2B* and activating mutations of *ALK* have been identified in NB families firstly (Trochet et al., 2004; Mosse et al., 2008). Furthermore, an increasing number of susceptibility genes have been observed in NB families, such as *NBPF1*, *NBPF23*, *BARD*, and *BRCA2* (Ritenour et al., 2018).

BRCA2 was first revealed to be involved in the homologous recombination (HR) repair pathway depending on interacting with the recombination protein RAD51. For DNA double-strand breaks (DSBs), HR is a high-fidelity mechanism of repair. As one of fifteen Fanconi anemia (FA) genes, BRCA2 also participates in the HR step in inter-strand crosslinks (ICLs) repair depending on RAD51. According to a previous study, about half of the cases of early-onset breast cancer are due to different inherited mutations in the *BRCA2* gene (Valencia et al., 2017). In pediatrics, two BRCA2 germline mutations associated with NB have been reported to date. One is BRCA2 p. W2830_E20splice detected from 56 children with NB (Zhang et al., 2015); the other is BRCA2 p. Y2215fs^∗^ identified from one NB family (Cai et al., 2017).

In this study, a new compound heterozygous mutation of the *BRCA2* gene was discovered in two siblings with PNTs by applying WGS technology. One of the biallelic mutations was *BRCA2* rs397507634 chr13: 32911104C > A (S871Ter), which was previously found from the screening of the BRCA1/2 genes (Alter, 2014). However, there are no reports on the relationship between this stop-gain mutant and diseases including PNTs. The other was a missense mutant *BRCA2* rs144848 chr13:32906729A > C (N372H). Although it was identified as highly related to breast cancer (Healey et al., 2000), ovarian cancer (Wenham et al., 2003), and other many kinds of cancers (Rudd et al., 2006; Ramus et al., 2008), there is no report on whether this missense mutant is associated with PNTs. The healthy parents harbored one different allele mutant of *BRCA2* separately. Unfortunately, the two children both inherited the two mutants from their parents and the novel compound heterozygous mutant of *BRCA2* led to PNTs. The evidence in this study may explain how both of the children suffer from the disease despite the absence of PNTs in their parents.

## Materials and Methods

### Patients and Samples

**FIGURE 1:**
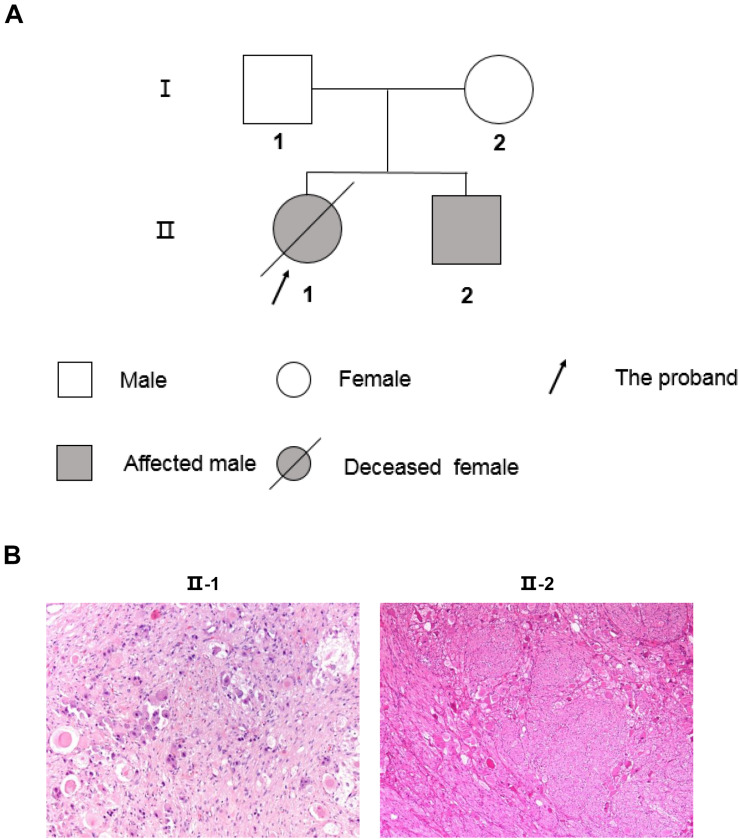
The two-generation pedigree of the family and pathological diagnosis of the PNTs siblings. **(A)** Solid symbols (squares = males, circles = females) indicate clinically affected individuals, and open symbols indicate unaffected individuals. **(B)** Histological study confirmed the type of PNTs. II-1 shows the immature neuroblastic tissue from NB after chemotherapy (H&E 100 ×). II-2 shows mature ganglion cells with abundant cytoplasm in a GN (H&E 50×).

This study included two young patients who were an elder sister (age at diagnosis was 30 months, II-1), and younger brother (age at diagnosis was 36 months, II-2), and their healthy parents (age at 30 s, I-1 and I-2), as shown in Figure 1A. All patients’ parents signed informed consent and this study obtained approval from the Institution’s Research Ethics Board of Hospital. DNeasy Blood and Tissue Kit (QIAGEN) was used for extracting genomic DNA from blood samples of the younger brother (II-2) and the parents (I-1 and I-2), and the tumor tissue of the siblings (II-1 and II-2). The elder sister’s tissue DNA was substituted for her genomic DNA from blood to analyze germline mutants accompanying other family members because her blood was unavailable because she had passed away when this study was conducted. DNA concentration was measured by Qubit^®^ DNA Assay Kit in Qubit^®^ 2.0 Fluorometer (Life Technologies, Carlsbad, CA, United States). RNA of the two patients’ tumor tissue was extracted by TRzol (Geng et al., 2019).

### Whole Genome Sequencing

A total of 0.5 μg of DNA per sample was used as input material for the DNA library preparations. Genomic libraries were prepared using the Illumina Truseq Nano DNA HT Sample Prep Kit following the manufacturer’s instructions. Libraries were analyzed for size distribution by Agilent 2100 Bioanalyzer. The clustering of the index-coded samples was performed on a cBot Cluster Generation System using Hiseq X PE Cluster Kit V2.5 (Illumina, San Diego, CA, United States) according to the manufacturer’s instructions. Then, the DNA libraries were sequenced on the Illumina Hiseq platform and 150 bp paired-end reads were generated.

### Read Mapping and Variant Calling

Reads after quality control were aligned to the UCSC human reference genome (GRCh37/hg19 assembly) using BWA 0.7.12-r1039 mem mode. Samtools-0.1.18 was used for sorting and removing PCR duplicates, and building an index for the bam files. Variants were called using the VarScan (version 2.3.9) pipeline (McKenna et al., 2010).

### Variant Annotation and Prioritization

The resulting variants were annotated and prioritized by ANNOVAR (Wang et al., 2010). A threshold of minor allele frequency (MAF < 0.01) from the 1000 Genomes Project Asian (Devuyst, 2015) and ExAC (Lek et al., 2016) non-TCGA cohorts was used to screen rare variants. The pathogenicity of rare missense variants was evaluated by SIFT (Li and Durbin, 2009), PolyPhen2 (Adzhubei et al., 2013), MutationTaster (Schwarz et al., 2014), and M-CAP (Jagadeesh et al., 2016; Li et al., 2019). Other rare variants, including frameshift, prematurely truncating, and initial codon variants were also retained for further analysis.

### Amplification and Sanger Sequencing of Mutation Sites

The two mutations in *BRCA2* were amplified by PCR with a Veriti 96-well Thermal Cycler (Applied Biosystems, Thermo Fisher Scientific). The primer sequences used were as follows: BRCA2-N372H (rs144848) F: 5′-CTGAAGTGGAACCAAATGATACTGA-3′, R: 5′-AGACGGTACAACTTCCTTGGAGAT-3′ Wenham et al., 2003); and BRCA2–S871X (rs397507634) F: 5′- AGTGGAATACAGTGATACTGAC -3′, R: 5′- TCGTTTACACAAGTCAAGTCTG -3′. Mutations were confirmed by Sanger sequencing (Bao et al., 2019).

### Differential Expression Analysis and KEGG Pathway Enrichment Analysis

RNA-seq data were aligned to the UCSC human reference genome (GRCh37/hg19 assembly) using Hisat2 2.0.1 (Kim et al., 2015). Expression of genes (raw count) was quantified by StingTie 1.3.5 (Pertea et al., 2015). Differentially expressed genes between different conditions were selected using R package DESeq2 based on the criteria of parameter: fold change > 2 and FDR < 0.05 (Love et al., 2014). KEGG pathway enrichment analysis of differentially expressed genes was carried out using the R package clusterProfiler (Yu et al., 2012). Only those pathways with adjusted *P*-value < 0.05 were considered statistically significant.

### Plasmids and Reagents

The p3xFlag-GV141-P/CAF plasmid was purchased from Shanghai GeneChem Co., Ltd. The fragment of BRCA2-290-453aa-wildtype was cloned from pDEST26 -BRCA2 gifted from Dr. Dongyi Xu (Peking University). Using BM seamless cloning kit (Biomed, China), the truncation was cloned into pXJ40-HA. The construct with a mutation in BRCA2-290-453aa-N372H was generated as described previously (Yang et al., 2015).

Anti-Flag M2 agarose affinity gel and mouse monoclonal antibody against Flag was purchased from Sigma (St. Louis, MO, United States). Antibody against HA and antibody against 53BP1 were from Abcam.

### Cell Culture and Reagents

SH-SY5Y cells were obtained from the American Type Culture Collection (Rockville, MD, United States). The cell line was grown in DMEM medium supplemented with 10% fetal bovine serum at 37°C in the presence of 5% CO2. For transient transfection experiments, cells were transfected with indicated constructs, using Lipofectamin 3000 (Invitrogen) following the manufacturer’s protocols.

### Coimmunoprecipitation and Western Blotting

SH-SY5Y cells transfected with p3xFlag-GV141-P/CAF and pXJ40-HA- BRCA2-290-453aa-WT or pXJ40-HA-BRCA2-290-453aa-N372H were harvested and immunoprecipitation with anti-Flag M2 agarose was performed using whole cell lysates as described previously (Wang et al., 2010). For the drug treatment group, 1 μM final concentration of Adriamycin (ADR) (Sigma) was added into the cell culture medium for 6 h before harvesting the whole cell lysis. Samples were separated by SDS-PAGE and detected by immunoblotting with indicated antibodies.

## Results

### Clinical Features

A 30-month-old Chinese girl (the proband, elder sister, and II-1; Figure 1) was diagnosed with NB, which primary site was retroperitoneum, in Shanghai Children’s Medical Center (SCMC) Affiliated to Shanghai Jiao Tong University. Serum neuron-specific enolase (NSE), serum lactate dehydrogenase (LDH), urinary vanillylmandelic acid (VMA), and urinary homovanillic acid (HVA) were all significantly elevated. The patient was diagnosed with high risk NB at stage M, with metastasis to bone and bone marrow, according to the international Neuroblastoma Risk Group (INRG) staging and risk system. Complying with SCMC-NB-2009 treatment protocol (Cai et al., 2017), the proband was treated with chemotherapy before removing the primary tumor. After surgery, the proband was determined to be NB by pathological examination (Figure 1B) according to international Neuroblastoma Pathology Classification (INPC). *MYCN* status, 1p36, and 11q23 were all normal without amplification or deletion. Although the myeloablative therapy, autologous stem cell transplant, radiation, and isotretinoin were following applied, the girl was recurrent after 26 months from the first diagnosis with NB. Then the proband accepted additional chemotherapy and allogeneic hematopoietic stem cell transplantation. Unfortunately, the patient died 53 months after the final onset of illness (Table 1).

**TABLE 1:** Clinical characteristics of two patients with peripheral neuroblastic tumors.

	Patient 1	Patient 2
	(Older, II 1)	(Younger, II 2)
Gender	Female	Male
Age at diagnosis (months)	30	36
Primary site	Retroperitoneal	Adrenal
Serum NSE	Elevated	Normal
Serum LDH	Elevated	Normal
Urinary VMA and HVA	Elevated	Normal
INRG stage	M (bone marrow, bone)	L1
INPC	Neuroblastoma	Ganglioneuroma
*MYCN* status	Not amplified	Not amplified
1p36	Normal	Normal
11q23	Normal	Normal
INRG risk	High	Very low
Treatment schedules	Chemotherapy, surgery, myeloablative therapy and autologous stem cell transplant, radiation, and isotretinoin	Surgery and observation
Recurrent time (months)	26	−
Rescue therapies	Additional chemotherapy, and allogeneic hematopoietic stem cell transplantation	−
Prognosis	Died of disease recurrence and progression	Alive without disease
Follow-up time (months)	53	So far

*NSE, neuron-specific enolase; LDH, lactate dehydrogenase; VMA, vanillylmandelic acid; HVA, homovanillic acid; INRG, International Neuroblastoma Risk Group; INPC, International Neuroblastoma Pathology Classification.*

The second case was the proband’s younger brother (63 months younger than his sister, II-2, and Figure 1). He was 36 months old at the time of diagnosis with GN, which is the same origin as NB but one benign type of PNTs in Beijing Children Hospital Affiliated with Capital Medical University. Unlike his sister, the tumor was in the adrenal gland. Serum NSE, serum LDH, urinary VMA, and urinary HVA were all normal. Besides, *MYCN* status, 1p36, and 11q23 were also normal without amplification or deletion, which was the same as his sister. The patient was diagnosed with very low risk GN at stage L1, according to the INRG staging and risk system. On account of this being a benign tumor, the treatment schedule only included surgery and observation, and no recurrence as yet (Table 1).

Information on the three generations of this family was collected. The grandfather of the proband died from liver cancer, and the grandmother is alive but suffering from gastric carcinoma and thyroid cancer. The maternal grandfather of the proband had Alzheimer’s, and the maternal grandmother lives with benign thyroid nodules. Other family members are healthy, including the father’s sister and her two children. The parents stated normal pregnancies and deliveries without any adverse event for the siblings.

### Whole-Genome Sequencing Analysis and Pathogenic Variants Validating

The fact that the siblings in the same family suffered from PNTs strongly suggested that their carcinogenesis was caused by germline variants. Therefore, to identify the causative variants contributing to the carcinogenesis, we performed high-coverage and high-quality WGS of all family members (father, mother, sister, and brother). Specifically, more than 91.4% of the whole genome had 10-fold coverage and showed high consistency among the experiments. In addition, more than 89% of sequenced bases reached Q_30_ based on the analysis of the Phred-scaled quality score (Supplementary Tables 1, 2).

**FIGURE 2:**
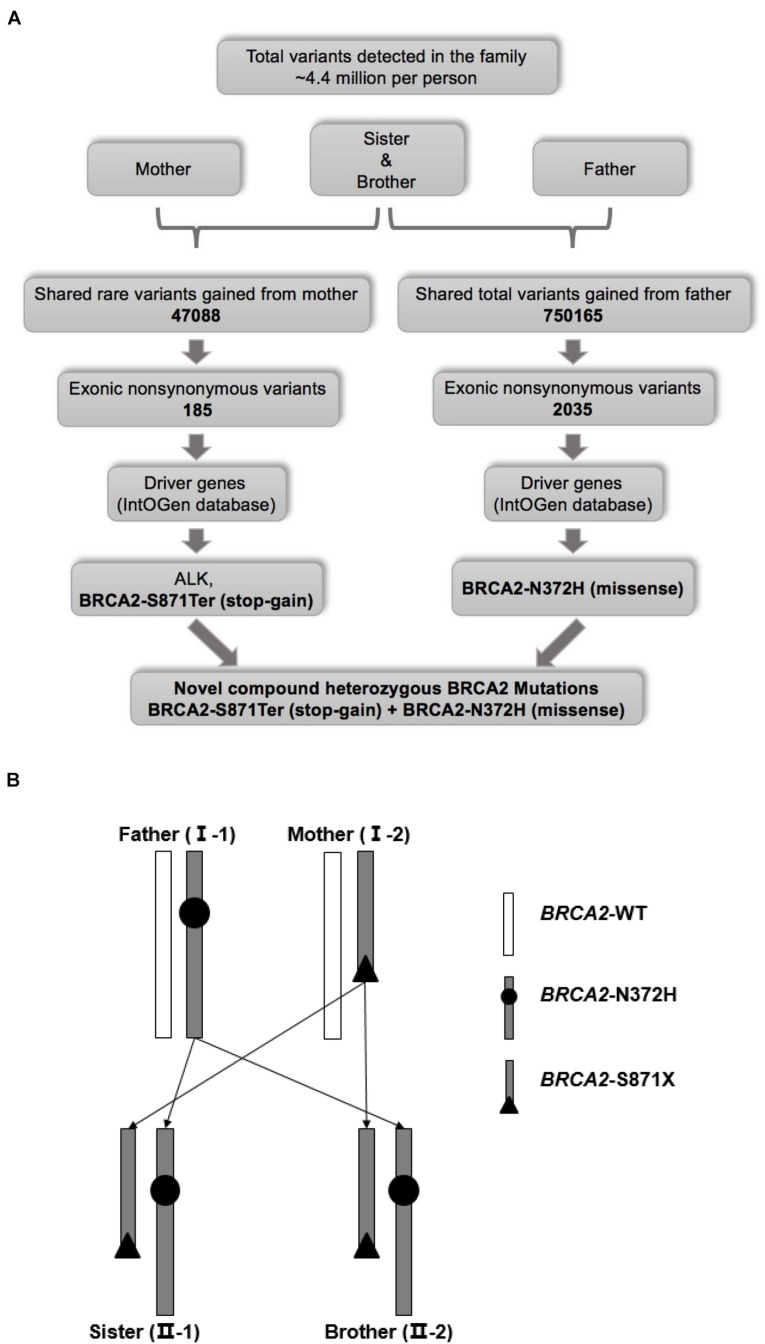
**(A)** Workflow for the identification of pathogenic mutations. **(B)** Schematic diagram of genetic pattern.

The variant calling analysis identified about 4.4 million variants in each family member (Supplementary Table 2). Of these, a total of shared 47088 rare variants of the siblings and their mother were selected based on MAF < 0.01. Furthermore, exonic non-synonymous variants were analyzed and 185 rare variants inherited from the mother were selected. Finally, among rare variants from the mother, as cancer driver genes, a missense mutant *ALK* (chr2: 30143039G > T, V163L, rs55697431), and a stop-gain mutant *BRCA2* (chr13: 32911104C > A, S871Ter, rs397507634) were focused through screening using the IntOGen database (Figure 2A). For ALK-V163L, multiple databases predicted that it was a benign mutation although *ALK* is one of the most famous susceptibility genes for NB (Supplementary Table 3).

**FIGURE 3:**
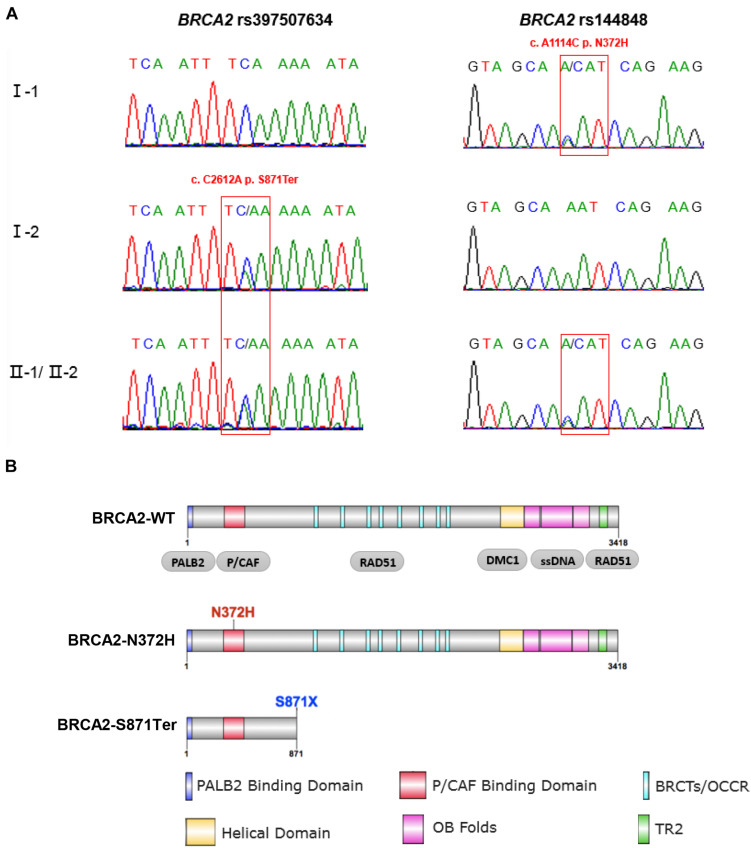
Sanger sequencing validation and functional domains of full-length and mutant BRCA2 protein. **(A)** Sanger sequencing confirmed the mutations of *BRCA2*. The two patients (II-1 and II-2) carried both of these two variants. The father carried BRCA2-N372H, while the mother carried BRCA2-S871Ter. **(B)** The full-length BRCA2 protein harbors six functional domains, including PALB2 binding domain, P/CAF binding domain, BRCTs repeat region, Helical domain, OB folds, and TR2 domain. BRCA2-N372H mutant is in the P/CAF binding domain; while the BRCA2-S871Ter variant results in premature truncation at the 871st amino acid of BRCA2 protein, which only includes the PALB2 and P/CAF binding domains.

Meanwhile, the same strategy was performed to select shared rare variants of the siblings and their father (Figure 2A). However, there was no same gene between the mother and father by this strategy (data not shown). Knudson’s “two-hit” theory, one susceptibility gene carried different mutants from mother and father separately. Further analysis revealed that they shared total variants gained from father (no MAF value limitation), and a missense mutant *BRCA2* (chr13:32906729A > C, N372H, rs144848) was selected. Although BRCA2-N372H is a common variant (C = 0.279642, GnomAD_exome) inherited from the father, it induced a novel compound heterozygous mutation of *BRCA2*, and combining with a stop-gain mutant BRCA2-S871Ter inherited from the mother. In other words, one allele was BRCA2-S871Ter (stop-gain) from the sibling’s mother, and the other was BRCA2-N372H (missense) from the father (Figure 2B and Supplementary Table 3). These two mutants of *BRCA2* were confirmed in the four persons by Sanger sequencing (Figure 3A). In a word, the novel compound heterozygous mutation *BRCA2* gene was locked as the cause of carcinogenesis.

### BRCA2-S871Ter Mutant Mechanistic Association With PNTs

As a stop-gain mutation, rs397507634 (S871Ter) in *BRCA2* was found first by quantitative polymerase chain reaction and high-resolution melting curve analysis (Coulet et al., 2010). This variation resulted in premature truncation at the 871st amino acid of the BRCA2 protein, which only includes the PALB2 and P/CAF binding domains (Figure 3B). Based on ClinVar annotation, rs397507634 in *BRCA2* is related to hereditary breast and ovarian cancer syndrome (RCV000590670.1), breast-ovarian cancer, familial 2 (RCV000077282.4), and hereditary cancer-predisposing syndrome (RCV000213349.1).

**FIGURE 4:**
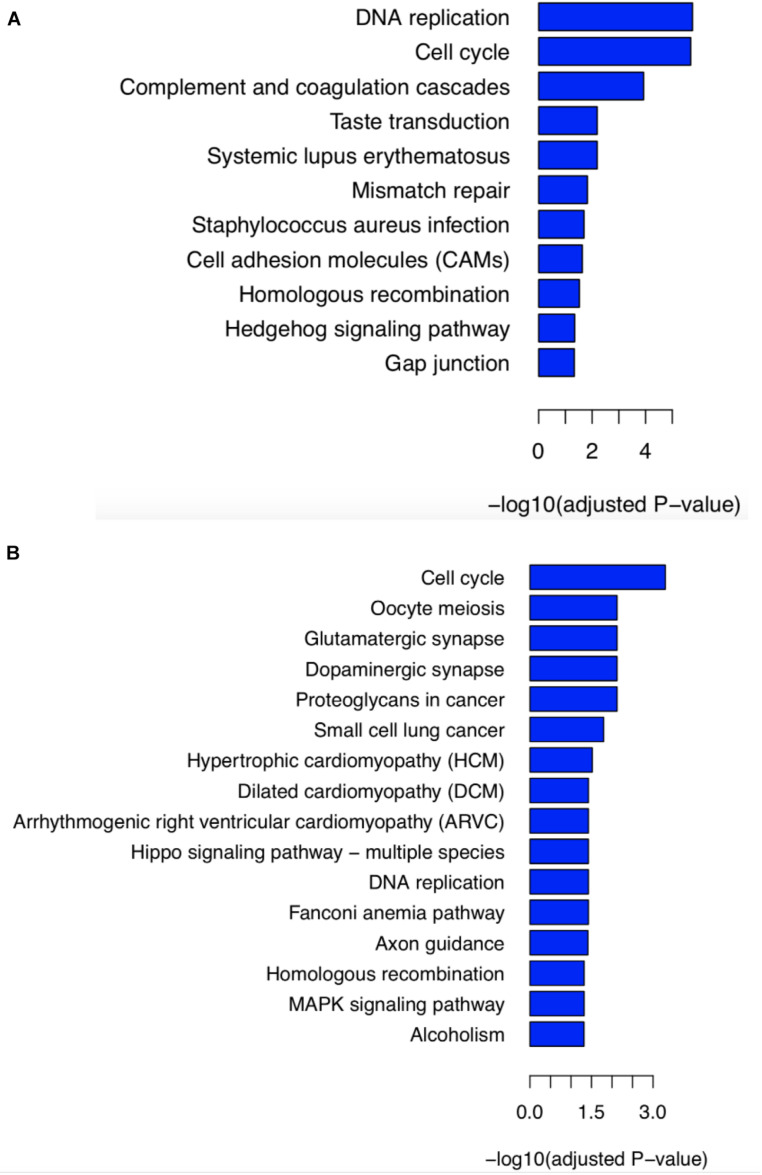
KEGG pathway enrichment analysis of differentially expressed genes. **(A)** To analyze the contribution of stop-gain mutant BRCA2-S871Ter, analysis based on differential genes obtaining from RNA-seq data was performed between siblings and four samples with BRCA2-N372H variant belonging to the GSE62564 database. **(B)** To reveal whether the compound heterozygous mutant of *BRCA2* contributed to the PNTs, we compared the siblings’ RNA-seq data, and 494 cases without either BRCA2-N372H or S871Ter mutant from the GSE62564 database.

By mining for the potential impact of stop-gain mutant BRCA2-S871Ter, differentially expressed genes were obtained from RNA-seq data of patients’ tumor tissue (Supplementary Table 4) and the GEO database GSE62564. There are four samples carrying BRCA2-N372H without BRCA2-S871Ter in the GSE62564 database, including three samples with homozygous mutant (NB_380, NB_390, and NB_438) and one sample with heterozygous mutant (NB_459). KEGG pathway enrichment analysis based on differential genes was performed between siblings and these four samples from the GSE62564 database. The result showed that the significant enriched KEGG pathway included DNA replication, cell cycle, and HR, which were identified with the molecular functions of BRCA2 (Figure 4A).

Besides, BRCA2-S871Ter led to early termination during BRCA2 protein translation and produced a 1–871 amino acid truncation of BRCA2 or even led to degradation of BRCA2. To simulate the functions of BRCA2-S871Ter, the GSE62564 dataset (498 samples) was analyzed. Samples with the top 25% (BRCA2 high group) and the lowest 25% (BRCA2 low group) expression of *BRCA2* were taken separately. KEGG pathway enrichment analysis based on these two groups was performed (Supplementary Figure 1). The results also revealed that pathways of the cell cycle, DNA replication, and HR were enriched significantly.

### BRCA2-N372H Mutant Mechanistic Association With PNTs

**FIGURE 5:**
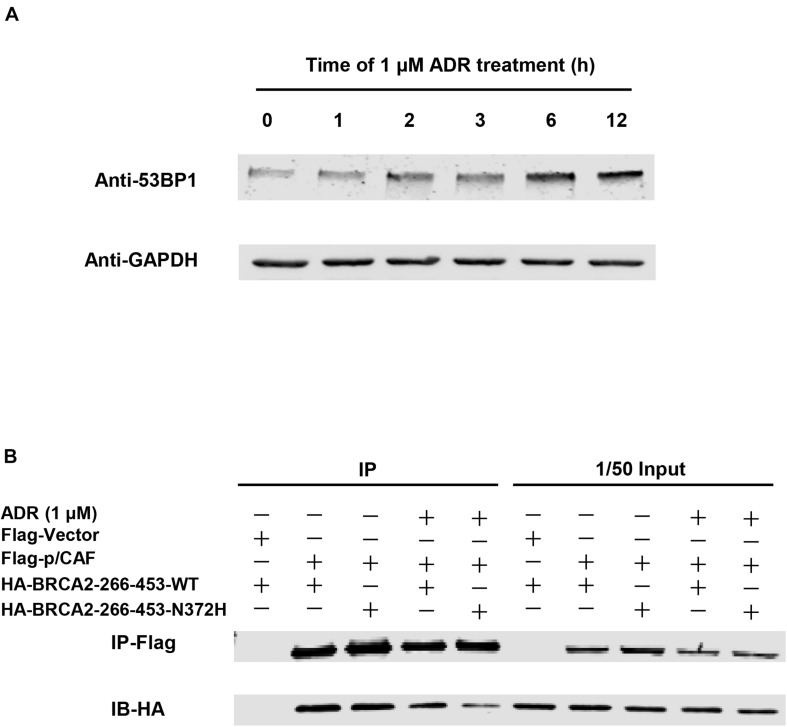
Co-IP between BRCA2 and P/CAF. **(A)** The optimum concentration and time of treatment with ADR in SH-SY5Y to induce DNA double-strand breaks. 1 μM ADR treated with 6 h could induce the expression of 53 bp1 enhanced significantly. **(B)** Co-IP showed that BRCA2-N372H substitution reduced the interaction between BRCA2 and P/CAF with the treatment of ADR (1 μM and 6 h). SH-SY5Y cells were transfected with plasmids expressing Flag-p/CAF and HA-BRCA2-266-453-WT or HA-BRCA2-266-453-N372H and analyzed by co-IP with or without ADR treatment. Immunoprecipitates were analyzed by western blot with antibodies against Flag or HA.

In addition to the BRCA2 premature truncating mutant, a missense variant rs144848 (N372H) of *BRCA2* inherited from proband’s mother was considered a pathogenic variant. It has been reported to increase the risk of many kinds of cancers, especially breast and epithelial ovarian, and indicating that the variant could increase cancer susceptibility. However, there was no report that BRCA2-N372H was associated with neuroblastic tumors. This mutant locates in the P/CAF binding domain (residues 290–453) of BRCA2 (Figure 3B). This domain has been shown to mediate the interaction between BRCA2 and the histone acetyltransferase P/CAF. The formation of the BRCA2-P/CAF complex is essential for the transcriptional activation of other genes (Fuks et al., 1998). Co-IP was performed and showed that BRCA2-N372H substitution did not affect the interaction between BRCA2 and P/CAF absenting chemotherapeutics drug (Figure 5B). To determine the optimum concentration and time of treatment with ADR, SH-SY5Y was treated with 1 μM ADR at a different time and then detected the expression of 53BP1 by WB. The results showed that 1 μM ADR treated for 6 h could induce the expression of 53BP1, which was enhanced significantly (Figure 5A). Remarkably, after the same condition of ADR treatment (1 μM, 6 h), the interaction between BRCA2 and P/CAF was reduced significantly due to the N372H mutant in the SH-SY5Y cell line (Figure 5B). The results suggest that the BRCA2-N372H mutant could destroy the function of P/CAF in the context of DNA damage.

### Dysfunctional BRCA2 Due to *BRCA2* Mutants

To reveal the compound heterozygous of *BRCA2* was contributed to PNTs’ carcinogenesis, KEGG pathway enrichment analysis was performed between the siblings’ RNA-seq data and 494 cases without either BRCA2-S871Ter or BRCA2-N372H mutants from the GSE62564 database. It was shown that the pathways of the cell cycle, DNA replication, FA pathway, and HR were enriched. (Figure 4B). It indicated that the compound heterozygous of *BRCA2* resulted in the disordering of BRCA2 protein’s function of DNA damage and response (DDR), which was the reason two child patients suffered PNTs.

To simulate the functions of the new compound heterozygous of *BRCA2*, the GSE62564 dataset (498 samples) was analyzed and the one intersection of samples with the top 25% expression of both *BRCA2* and *P/CAF* (BRCA2-P/CAF high group, 36 samples) was taken. Meanwhile, samples with the lowest 25% expression of both *BRCA2* and *P/CAF* were taken as the other intersection from GSE62564 (BRCA2-P/CAF low group, 39 samples). KEGG pathway enrichment analysis was performed between the two intersections (Supplementary Figure 2). The results also revealed that pathways of the cell cycle, DNA replication, FA pathway, and HR pathways were enriched significantly, which was similar to our previous results (Figure 4B).

## Discussion

*BRCA2*, a principal tumor suppressor gene, is involved in DNA damage repair and related to many types of cancer susceptibility, especially breast cancer, and ovarian cancer in adults (Rousset-Jablonski and Gompel, 2017; Flaum et al., 2019). Meanwhile, as one of the FA DNA repair-related genes, some specific *BRCA2* mutants lead to a severe subset FA accompanying the early onset of cancer, including acute myeloid leukemia, brain tumors, Wilms tumor, and so on (Alter, 2014). In pediatrics, BRCA2 p. W2830_E20splice and p. Y2215fs^∗^ are the only two *BRCA2* germline mutations to have been reported to associate with NB (Zhang et al., 2015; Cai et al., 2017).

This study revealed a new compound heterozygotes mutant of *BRCA2* by employing WGS of germline DNA in two siblings with PNTs and their unaffected parents. A stop-gain mutant BRCA2-S871Ter inherited from the mother and a missense mutant BRCA2-N372H inherited from the father were validated by Sanger sequencing (Figure 3A). According to Knudson’s “two-hit” theory (Knudson, 1996) and KEGG pathway enrichment analysis (Figure 4B and Supplementary Figure 2), our results provide useful information that this novel compound heterozygotes mutant of *BRCA2* could be the cause of carcinogenesis and a potential biomarker for PNTs.

For the stop-gain mutant, inherited from the mother of the siblings, BRCA2-S871Ter is a rare frequency variation, which has been found through qPCR-HRM based on 210 patients (Coulet et al., 2010). Moreover, this variation is included in some panels to detect breast cancer or ovarian cancer in clinical settings. It can lead to early termination during BRCA2 protein translation and produce a 1–871 amino acids truncation of BRCA2. In this study, western blotting was performed to detect BRCA2 expression in the siblings’ tumor tissue. Unfortunately, there was no specific signal of BRCA2 in WB (data not shown). The reason for failing to detect BRCA2 might be protein degradation for its large molecular weight. Although it is not certain whether this truncation is expressed or degraded after translation, the fact is that the functions of BRCA2 should be impacted to a great extent. As Figure 3B shows, the majority of the important domains, including BRCTs domain, helical domain, OB folds, and the TR2 domain, are not in the BRCA2 stop-gain truncation. These domains all play key roles in DNA double strand breaks repair. The BRCTs and OB fold domain are essential to BRCA2 binding to RAD51. Then the BRCA2-RAD51 complex will further promote RAD51 assembly onto single-strand DNA to carry forward HR repair. KEGG pathway enrichment analysis also confirmed the above results about BRCA2-S871Ter (Figure 4A and Supplementary Figure 1).

In addition, BRCA2-N372H is a variant associated with breast cancer and epithelial ovarian cancer (Healey et al., 2000; Wenham et al., 2003). Recently, an increasing number of studies have been performed to prove that the BRCA2-N372H variant is related to susceptibility to many other cancers, including multiple lymphoma, prostate cancer, advanced esophageal squamous cell carcinoma, familial colorectal tumors, and so on. Moreover, previous research has shown that BRCA2-N372H is located in the P/CAF binding domain of BRCA2. The domain from residues of amino acid 290–453 in the N-terminus of BRCA2 specifically interacts with the histone acetyltransferase P/CAF (Fuks et al., 1998). P/CAF belongs to the type three family of lysine acetyl transferases (KAT3) (Goodman and Smolik, 2000). As a transcriptional co-activator, P/CAF binds to transcription factors and performs lysine acetyltransferases activity to acetylate these transcription factors and histones to lose condensed chromosomes. N terminus of BRCA2 is equipped with HAT activity when it bounds to P/CAF. The BRCA2-N372H variation weakens BRCA2 expression and BRCA2-P/CAF interaction and further reduces sensitivity to paclitaxel in breast cancer cells (Kwon et al., 2017). It indicates that BRCA2 372-His substitution induces sequential changes of BRCA2-P/CAF interaction and HAT activity, which is important in paclitaxel resistance (Kwon et al., 2017). ADR as the first-line chemotherapy drug to NB therapy could induce DSBs to NB cells. In our study, with ADR treatment, the interaction between BRCA2, and P/CAF was reduced significantly due to the N372H mutant in SH-SY5Y cells (Figure 5). Meanwhile, BRCA2-N372H substitution could not affect the interaction between BRCA2 and P/CAF without drugs. The results suggested that the BRCA2-N372H mutant could destroy the HAT activity of BRCA2-P/CAF in the context of DNA damage. Furthermore, like the paclitaxel resistance, this mutant could induce ADR resistance to NB cells due to lack of HAT activity. This indicates that the BRCA2-N372H mutant could be a new potential drug target in NB chemotherapy.

This study explored the reason for different malignant degrees between these two child patients. Firstly, the BRCA2-N372H variant was reported to affect fetal survival in a sex-dependent manner. With the same BRCA2-N372H variant, the survival rate of newborn females was significantly lower than males (Healey et al., 2000). Secondly, WGS of the two children’s tumor tissue did not show enrichment of any distinct carcinogenesis related pathway in specific somatic variants in the siblings, respectively (Supplementary Figure 2). There were 721 individual mutant genes in the elder sister and 701 individual mutant genes in the brother. However, screening the IntOGen database revealed that the elder sister harbored more specific cancer driver genes than a brother (20 vs. 14 genes), indicating that the sister had a greater chance of developing a malignant tumor (Supplementary Tables 6,7). Furthermore, RNA-seq analysis based on the two siblings’ tissue RNA revealed that the row counts of *BRCA2* mRNA expression were 91 in the sister and 898 in the brother (Supplementary Table 5). Real-time PCR was performed to confirm the relative expression of *BRCA2* in the siblings’ tumor tissue. It was verified that the expression of *BRCA2* in the sister was significantly lower than in the younger brother (Supplementary Figure 3). Although the sample size was limited in this study, the siblings harbored similar genetic backgrounds and the same compound heterozygous mutation of *BRCA2*. This indicated that as tumor suppressor genes, a low amount of BRCA2 may lead to cancer susceptibility, and therefore the malignancy degree in the sister was higher than the younger brother. However, the mechanism of different malignant degrees between the siblings needs further study.

## Conclusion

Our data revealed that a novel compound heterozygous mutation of the *BRCA2* gene is associated with PNTs by disordering DDR signal pathway. One allele was stop-gain mutant BRCA2-S871Ter, inherited from their mother. The other was a missense mutant BRCA2-N372H inherited from their father, which was confirmed to impair the interaction between BRCA2 and P/CAF, and induced ADR resistance in NB. Moreover, these results provided potential laboratory evidence for the clinical and prenatal diagnosis of PNTs. These results could also potentially guide clinical precision medication. For further investigation of the genotype-phenotype relationship in PNTs, future efforts should be made to recruit more families affected by this disease, and despite its rarity.

## Limitations

There were some limitations in the present study. Firstly, the sample size was small, due to the study object was only one family with two siblings harboring PNTs. Secondly, we needed to make a credible conclusion by incorporating prior knowledge, because of the rarity of samples. Therefore, to further investigate the genotype-phenotype relationship in PNTs, efforts should be made to recruit more families affected by this disease. Furthermore, to decipher the molecular mechanism of the compound heterozygous of *BRCA2* mutations, more studies, including cell level and animal models, and need to be performed in the future.

## Data Availability Statement

The datasets presented in this study can be found in online repositories. The names of the repository/repositories and accession number(s) can be found below: https://www.biosino.org/node/analysis/detail/OEZ005703, OEZ005703.

## Ethics Statement

The studies involving human participants were reviewed and approved by Ethics Committee of the Beijing Children’s Hospital, Capital Medical University. Written informed consent to participate in this study was provided by the participants’ legal guardian/next of kin. Written informed consent was obtained from the individual(s), and minor(s)’ legal guardian/next of kin, for the publication of any potentially identifiable images or data included in this article.

## Author Contributions

YrY, JC, and HQ conducted the experiments, analyzed the data, and wrote the manuscript. YJ and LZ analyzed the data, reviewed, and edited the manuscript. SY, HW, LF, EH, YbY, JL, YC, and XN conducted the experiments. MX, TS, and YG designed the study, wrote, reviewed, and edited the manuscript, and administrated the project. All authors contributed to the article and approved the submitted version.

## Conflict of Interest

The authors declare that the research was conducted in the absence of any commercial or financial relationships that could be construed as a potential conflict of interest.

## Publisher’s Note

All claims expressed in this article are solely those of the authors and do not necessarily represent those of their affiliated organizations, or those of the publisher, the editors and the reviewers. Any product that may be evaluated in this article, or claim that may be made by its manufacturer, is not guaranteed or endorsed by the publisher.

## Supplementary Material

Supplementary Figure 1 KEGG pathway enrichment analysis of differentially expressed genes between BRCA2 high group and BRCA2 low group from GSE62564 dataset.

Supplementary Figure 2 KEGG pathway enrichment analysis of differentially expressed genes between BRCA2-P/CAF high group and BRCA2-P/CAF low group from GSE62564 dataset.

Supplementary Figure 3 KEGG pathway enrichment analysis of specific somatic variants. **(A)** KEGG pathway enrichment of 721 individual mutant genes in elder sister. **(B)** KEGG pathway enrichment of 701 individual mutant genes in brother.

Supplementary Figure 4 Expression of *BRCA2* in the siblings. Real-time PCR to detect expression of *BRCA2* in the siblings’ RNA from tumor tissue.
