# Supplementary figures and images for: A Novel Germline Compound Heterozygous Mutation of BRCA2 Gene Associated With Familial Peripheral Neuroblastic Tumors in Two Siblings
Source: Front Genet. 2021 Jul 23;12:652718. doi: 10.3389/fgene.2021.652718 (PMC8343186; doi:10.3389/fgene.2021.652718)

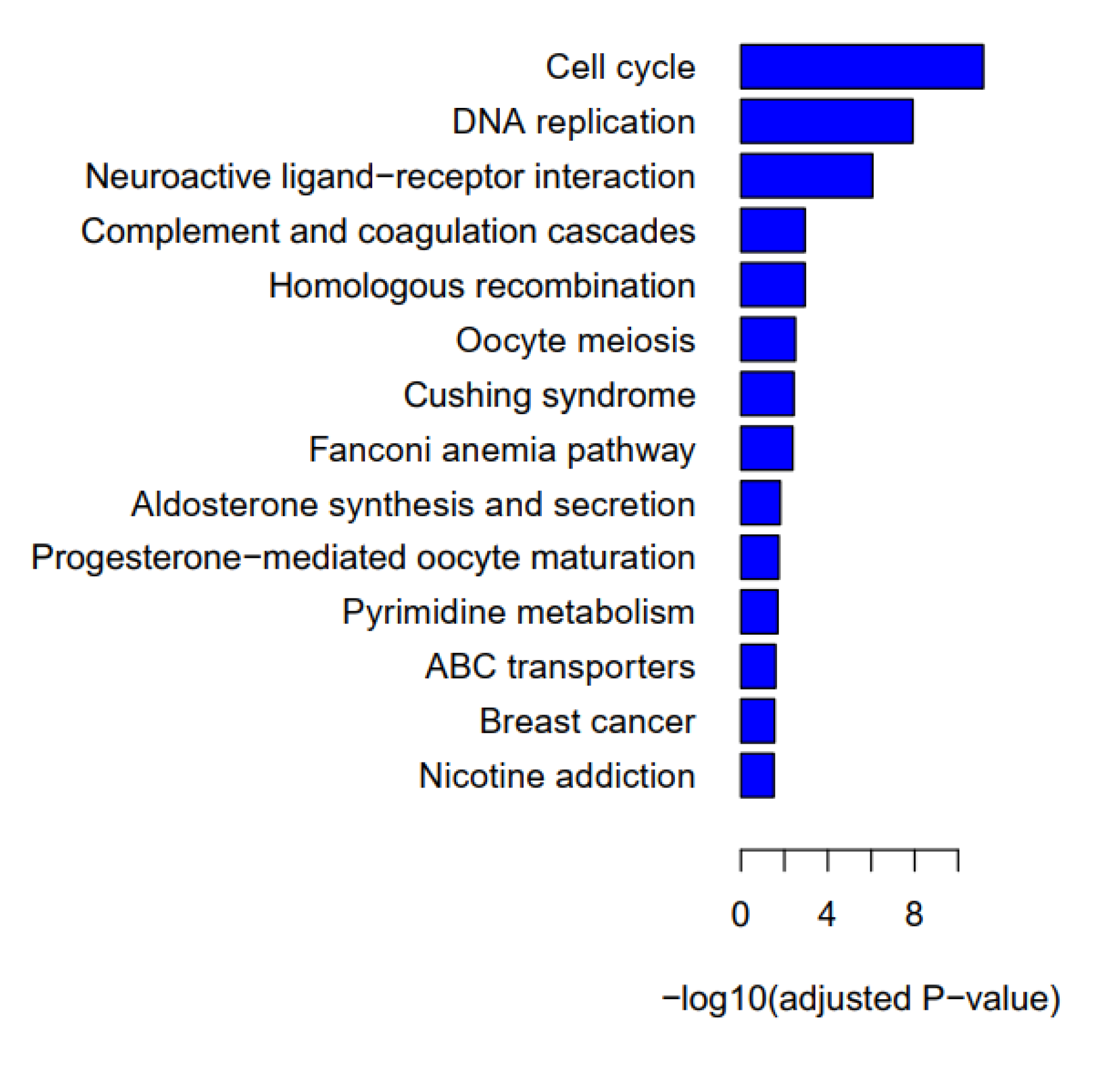

Supplement: Supplementary Figure 1 — KEGG pathway enrichment analysis of differentially expressed genes between BRCA2 high group and BRCA2 low group from GSE62564 dataset. [file Image_1.TIF]

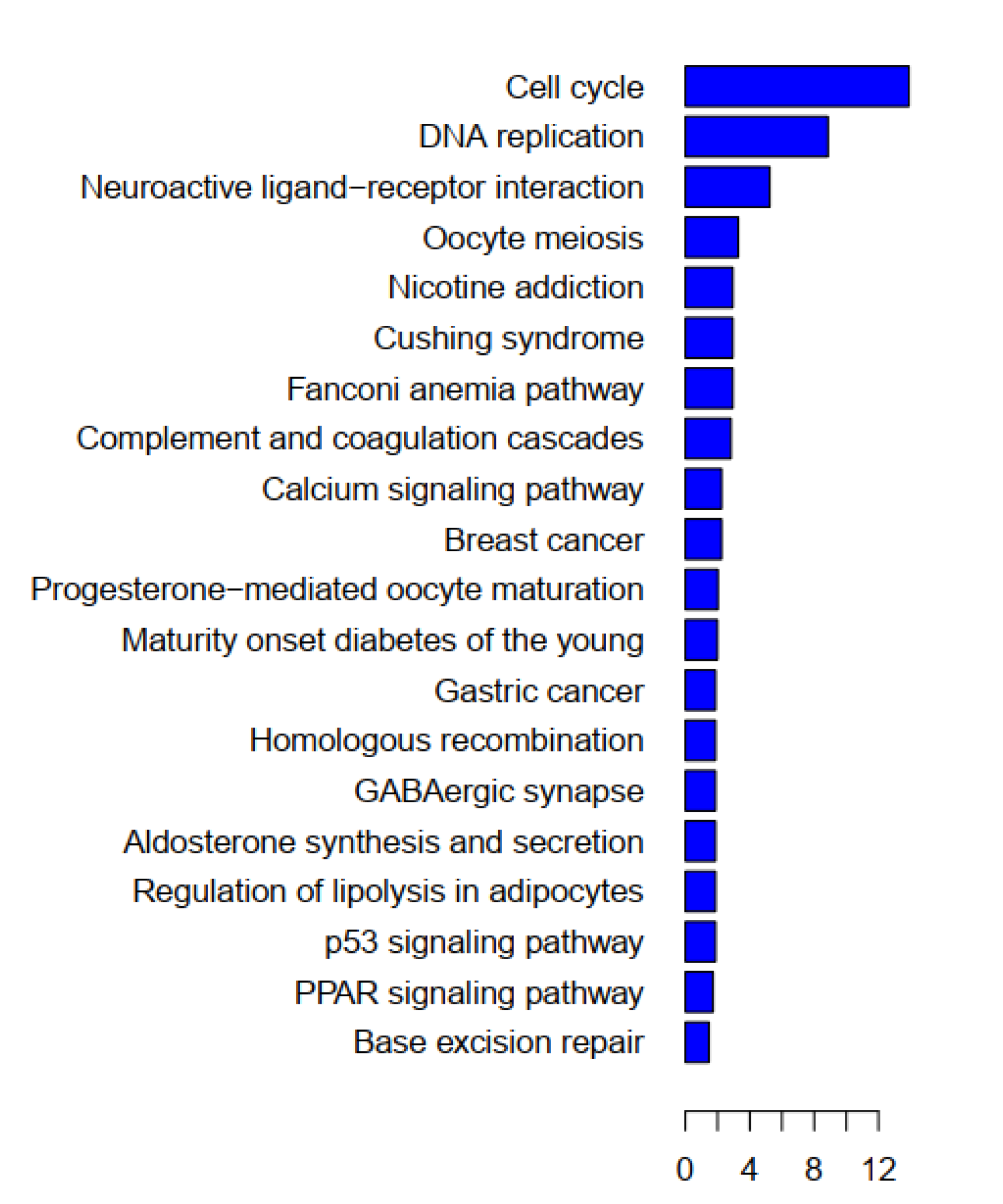

Supplement: Supplementary Figure 2 — KEGG pathway enrichment analysis of differentially expressed genes between BRCA2-P/CAF high group and BRCA2-P/CAF low group from GSE62564 dataset. [file Image_2.TIF]

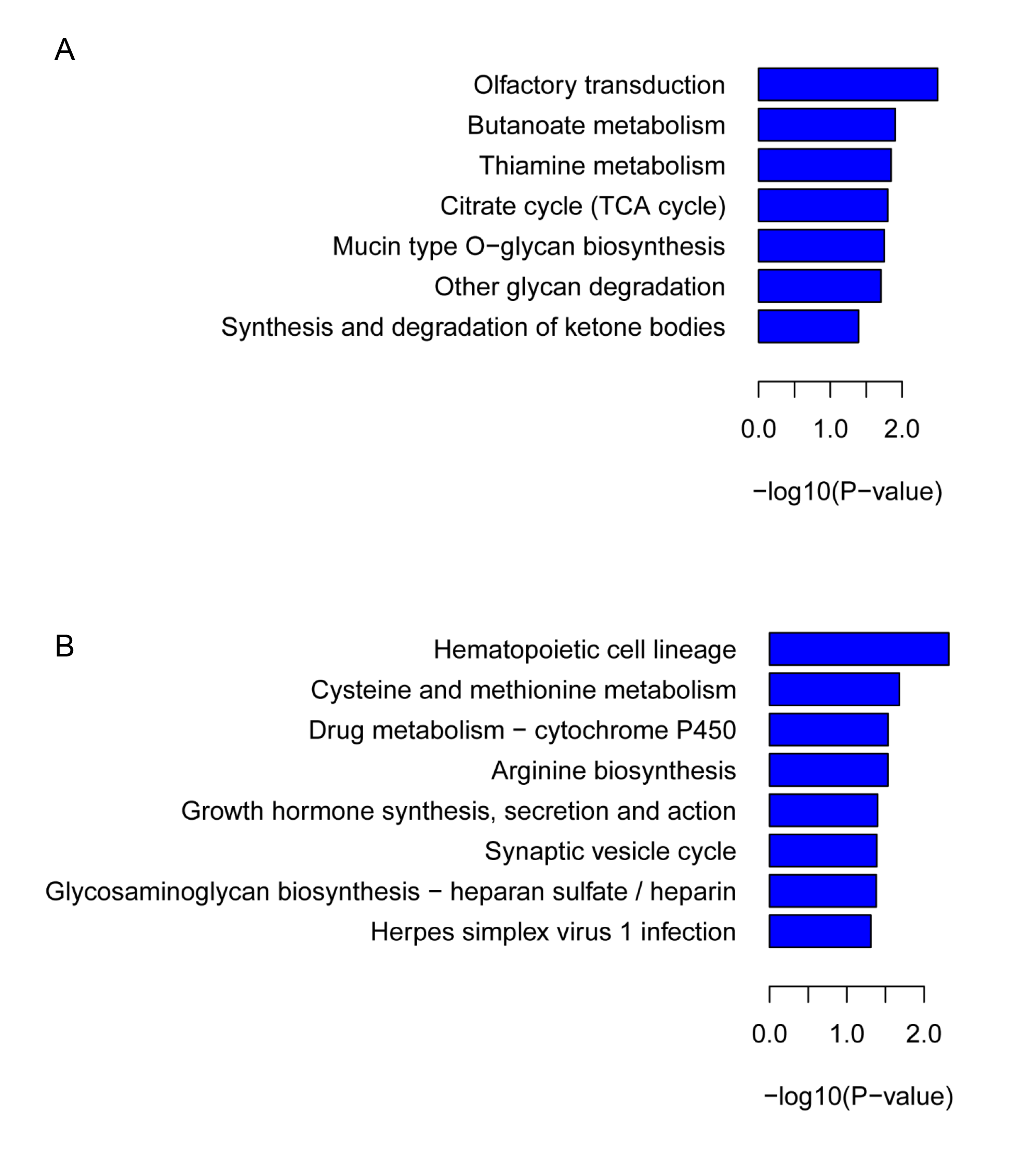

Supplement: Supplementary Figure 3 — KEGG pathway enrichment analysis of specific somatic variants. (A) KEGG pathway enrichment of 721 individual mutant genes in elder sister. (B) KEGG pathway enrichment of 701 individual mutant genes in brother. [file Image_3.TIF]

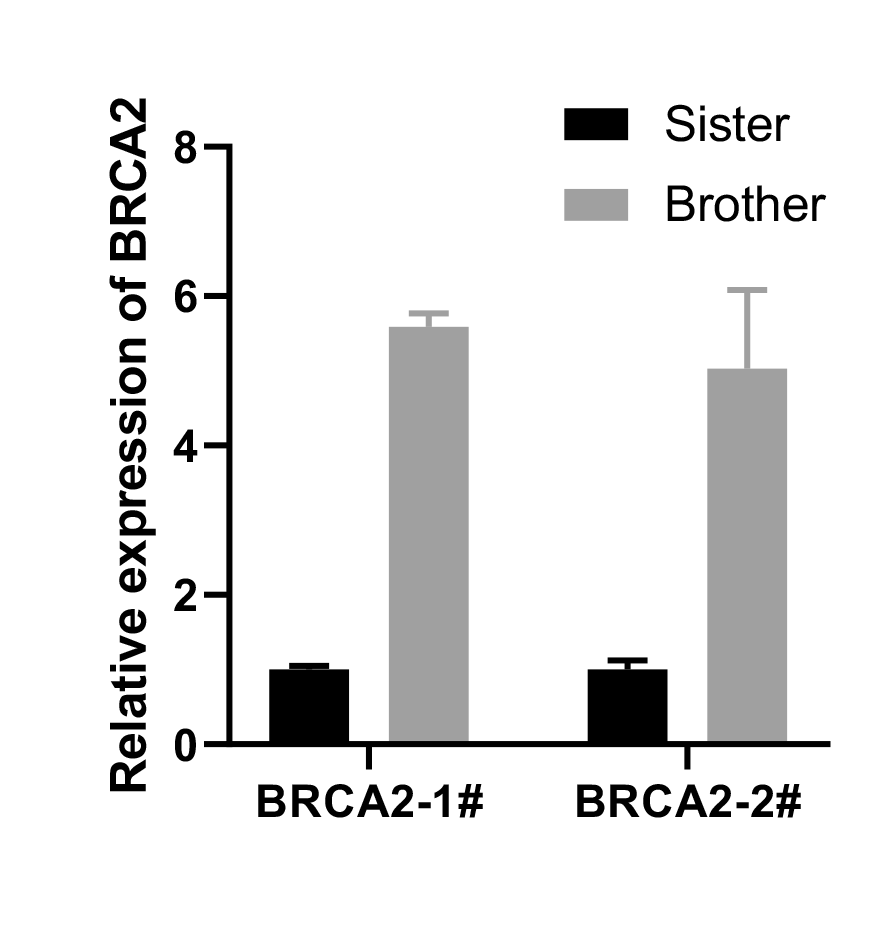

Supplement: Supplementary Figure 4 — Expression of BRCA2 in the siblings. Real-time PCR to detect expression of BRCA2 in the siblings’ RNA from tumor tissue. [file Image_4.TIF]
